# Peptide GTSFTTTAER From *Rapana venosa* Alleviates TNBS‐Induced Inflammatory Bowel Disease in a Zebrafish Model via Multi‐Pathway Regulation

**DOI:** 10.1002/fsn3.70427

**Published:** 2025-06-12

**Authors:** Qifei Wang, Fenghua Xu, Yongna Cao, Bin Li, Shanshan Zhang, Yun Zhang

**Affiliations:** ^1^ Biology Institute Qilu University of Technology (Shandong Academy of Sciences) Jinan China; ^2^ Research Center of Translational Medicine Central Hospital Affiliated to Shandong First Medical University Jinan China

**Keywords:** inflammatory bowel disease, peptide GTSFTTTAER, *Rapana venosa*, toll‐like receptor, zebrafish

## Abstract

Inflammatory bowel disease (IBD) is a refractory chronic intestinal disease caused by immune dysfunction, with an unknown pathogenesis. In this study, the peptide GTSFTTTAER was isolated from *Rapana venosa* for treatment of IBD for the first time. We examined its protective effects on a zebrafish model of 2,4,6‐trinitrobenzene sulfonic acid (TNBS)‐induced IBD. The results indicate that the peptide GTSFTTTAER ameliorates intestinal inflammatory injury by reducing the number of immune cells at the intestinal site and increasing the frequency of intestinal peristalsis. Besides, in order to predict and verify the potential mechanism of the anti‐inflammatory effects of peptide GTSFTTTAER, we performed transcriptome and reverse transcription‐quantitative polymerase chain reaction analysis. The transcriptome analysis revealed that the key pathways for the potential protective effects of GTSFTTTAER were the Toll‐like receptor signaling pathway and the necroptosis pathway. Lastly, molecular docking technology further confirmed the action target of peptide GTSFTTTAER. In conclusion, GTSFTTTAER has a beneficial effect on IBD in TNBS‐induced zebrafish. Our study will provide a valuable reference for the utilization of peptide GTSFTTTAER from *Rapana venosa*, and it may also be helpful in developing therapeutic agents for IBD.

## Graphical abstract

The peptide GTSFTTTAER from Rapana venosa significantly alleviates intestinal inflammation in a zebrafish model of TNBS‐induced inflammatory bowel disease by reducing immune cell infiltration and enhancing intestinal motility. Transcriptome and molecular docking analyses reveal that GTSFTTTAER's protective effects are mediated through Toll‐like receptor signaling and necroptosis pathways, establishing its potential as a therapeutic agent for IBD.

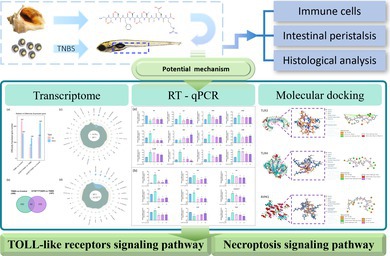

**Abbreviations**
5‐ASA: 5‐aminosalicylic acid
AB: alcian blue staining
DEG: differentially expressed gene
dpf: day post fertilization
GO: gene ontology
H&E: Hematoxylin and Eosin
IBD: inflammatory bowel disease
IEE: intestinal efflux efficiency
IOD: integrated option density
KEGG: Kyoto encyclopedia of genes and genomes
RT‐qPCR: reverse transcription‐quantitative polymerase chain reaction
TEM: transmission electron microscope
TLR: Toll‐like receptor
TNBS: 2,4,6‐trinitrobenzene sulfonic acid

## 1 Introduction

Inflammatory bowel disease (IBD) is characterized by systemic, autoimmune, and chronic inflammation affecting the colon and small intestine, encompassing ulcerative colitis and Crohn's disease. These conditions may result in severe complications, including colorectal cancer (Marotti et al. 2024; Zhou et al. 2022). Although the incidence of IBD has stabilized in Western countries, its prevalence continues to increase in developing countries, presenting a significant challenge to healthcare systems (Dong et al. 2022). Despite the clinical use of anti‐inflammatory agents, immunosuppressants, and biologic therapies for IBD treatment, long‐term administration of these drugs can lead to adverse effects such as autoimmune responses, viral infection, and tumorigenesis (Tsujii et al. 2022; Zhang et al. 2022). Consequently, the development of more effective and safer therapeutic strategies for IBD remains a critical priority.

Recent studies have highlighted the potential benefits of certain nutrients, food components, and bioactive peptides (Deng et al. 2020). In exploring novel IBD therapies, bioactive peptides have garnered substantial attention due to their advantageous properties, including excellent cell diffusion, high permeability, small molecular size, low toxicity, and cost‐effectiveness (Li, Yuan, et al. 2022). Bioactive peptides with anti‐inflammatory, antioxidant, immunomodulatory, and prebiotic attributes have been shown to support intestinal homeostasis (Fernandez‐Tome et al. 2019). Additionally, there is growing interest in marine‐derived bioactive peptides, driven by the increasing exploration and utilization of marine resources (Ucak et al. 2021). Among these, *Rapana venosa*, a predatory marine snail belonging to the Muricidae family, has drawn attention for its bioactive compounds with antioxidant and anti‐inflammatory activities (Prelipcean et al. 2022).

In this study, we focus on GTSFTTTAER (Gly‐Thr‐Ser‐Phe‐Thr‐Thr‐Thr‐Ala‐Glu‐Arg), a novel peptide isolated and purified from the visceral mass of *Rapana venosa*. This peptide has a molecular weight of 1.1 kDa and exhibits unique structural features, such as a balanced composition of hydrophobic and polar residues. Compared to terrestrial‐derived anti‐inflammatory peptides (e.g., lactoferrin‐derived peptides or soybean peptides), GTSFTTTAER demonstrates three distinct advantages: (1) its smaller molecular size enhances intestinal permeability; (2) its compact tertiary structure confers resistance to enzymatic degradation; and (3) its marine origin reduces the risk of immunogenicity, providing new insights into IBD therapeutics.

To evaluate the therapeutic potential of GTSFTTTAER, we utilized a zebrafish ( *Danio rerio* ) model of TNBS‐induced IBD. The compound 2,4,6‐trinitrobenzene sulfonic acid (TNBS), which induces intestinal cell injury and inflammation, is widely used to establish IBD models in mammals (Kamareddine et al. 2020). Due to their genetic tractability, external development, and high reproductive capacity during embryonic and early larval stages, zebrafish ( *Danio rerio* ) are extensively employed in human disease research and activity screening (Huang et al. 2021). A key advantage of zebrafish is the ability to fluorescently label innate immune cells, enabling direct observation of immune cell dynamics in living larvae due to their optical transparency (Buchan et al. 2019; López Nadal et al. 2020). The migration and aggregation of immune cells serve as markers reflecting inflammation. For these reasons, zebrafish are considered an excellent model organism for studying intestinal physiology and disease. In the present investigation, we assessed the anti‐inflammatory effect of the peptide GTSFTTTAER against TNBS‐induced intestinal inflammation in zebrafish and further explored the underlying mechanisms.

## 2 Materials and Methods

### 2.1 Chemicals and Reagents

**FIGURE 1:**
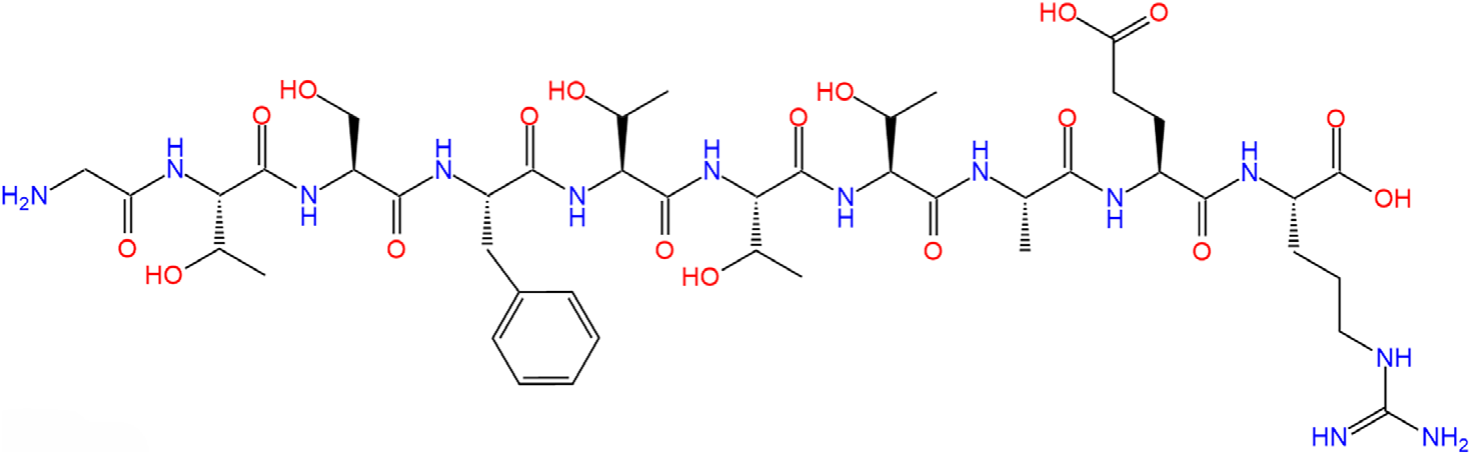
The 2D structure of peptide GTSFTTTAER.

The peptide GTSFTTTAER (Figure 1) used in the study was synthesized via solid‐phase synthesis technology by CelLmano Biotechnology Co. Ltd., Hefei, China, with a purity with the assistance of OE Biotecof 95%. TNBS, 5‐aminosalicylic acid (5‐ASA), and calcein were purchased from Sigma‐Aldrich (Shanghai, China). The remaining chemicals and reagents were of analytical grade and were purchased from local commercial sources. The SPARKeasy RNA isolation kit and the SPAPKscript II RT Plus Kit were purchased from Sparkjade (Qingdao, China).

### 2.2 Animals and Housing

Animal experiments were performed using wild‐type zebrafish and transgenic zebrafish Tg (*zlyz*: EGFP) with enhanced expression of green fluorescent protein labeling of inflammatory cells. These zebrafish were obtained from the Engineering Research Center of Zebrafish Models for Human Diseases and Drug Screening of Shandong Province. The zebrafish were maintained in an environment with a constant temperature (28°C ± 0.5°C) and a light/dark cycle (14 h:10 h). Healthy female zebrafish were paired with male zebrafish at a ratio of 2:2 overnight. The next morning, the embryos were collected and disinfected with a methylene blue solution. Then, the eggs were placed in zebrafish embryo culture water containing 5 mM NaCl, 0.17 mM KCl, 0.33 mM CaCl_2_, and 0.33 mM MgSO_4_. Dead eggs were removed every 24 h, and the embryo medium was replaced with fresh medium.

### 2.3 Experimental Grouping and Treatment

In this experiment, the zebrafish were divided into six groups: the blank control group; the TNBS‐induced model group (50 μg/mL); the 5‐ASA positive control group (50 μg/mL TNBS +6.5 μM 5‐ASA); and the low, medium, and high‐dosage GTSFTTTAER groups (50 μg/mL TNBS +25 μM GTSFTTTAER, 50 μg/mL TNBS +50 μM GTSFTTTAER, 50 μg/mL TNBS +100 μM GTSFTTTAER). 5‐ASA is a clinically available drug for the treatment of IBD, so 6.5 μM 5‐ASA was selected as a positive control drug (Cao et al. 2024). Zebrafish at 3 days post‐fertilization (dpf) were randomly transferred to 24‐well plates, each well containing 2 mL of bathing medium. Three replicate wells were set up for each group, with 10 zebrafish in each well. Then, except for the control group, the zebrafish were exposed to 50 μg/mL TNBS for 48 h to induce IBD‐like conditions (Xu et al. 2024). After that, the IBD zebrafish larvae were immersed in different concentrations of GTSFTTTAER (25, 50, and 100 μM) and 6.5 μM 5‐ASA for 24 h.

### 2.4 Detection of Intestinal Immune Cell Aggregation in Zebrafish

The experimental animals were Tg (*zlyz*: EGFP) zebrafish, which were grouped and treated in the same way as in section 2.3. After drug treatment, the zebrafish were anesthetized with tricaine (0.2%, w/v), fixed laterally, and then photographed using a fluorescent microscope (Olympus, SZX2‐ILLTQ, Tokyo, Japan). The number of aggregated immune cells in the intestinal area was counted and used as an indicator for evaluating inflammation.

### 2.5 Determination of Intestinal Efflux Efficiency

After the TNBS‐induced IBD model was successfully established, the zebrafish in each group were stained with a 0.2% calcein solution for 1.5 h. After staining, the zebrafish were rinsed with phosphate‐buffered saline and photographed under an Olympus SZX16 fluorescence microscope (Tokyo, Japan). The average integrated optical density (IOD) of the control group was defined as IOD_0_, and the average IOD of the TNBS group was defined as IOD_1_. After 16 h of incubation with 5‐ASA and the peptide GTSFTTTAER, the fluorescence intensity of the zebrafish gut was captured using the fluorescent microscope, and the IOD was determined (IOD_2_). Finally, the IOD was analyzed by Image Pro‐Plus 5.1, and the intestinal efflux efficiency (IEE) was calculated using the equation proposed by Wang et al. (2023).

$$\mathrm{IEE}\;\left(\text{control group}\right)=\frac{{\mathrm{IOD}}_{0}-{\mathrm{IOD}}_{2}}{{\mathrm{IOD}}_{0}}$$

$$\mathrm{IEE}\;\left(\text{Other groups}\right)=\frac{{\mathrm{IOD}}_{1}-{\mathrm{IOD}}_{2}}{{\mathrm{IOD}}_{1}}$$

### 2.6 Frequency of Intestinal Peristalsis

In the experiment to assess the promoting effect on intestinal peristalsis, healthy wild‐type zebrafish were selected. The grouping and sample dosages were consistent with those in section 2.3. After the drug treatment, the zebrafish were stained with a 0.2% calcein solution for 1.5 h. After being washed three times with phosphate‐buffered saline, the number of intestinal peristaltic movements of the stained zebrafish in 1 min was recorded under a fluorescence microscope.

### 2.7 Histological Analysis

After drug treatment, 10 zebrafish larvae were randomly selected from each group. They were fixed in 4% paraformaldehyde for 24 h and then embedded in paraffin. Hematoxylin and Eosin (H&E) staining and Alcian Blue (AB) staining were carried out on the zebrafish intestinal tissue. In addition, another 10 zebrafish larvae were randomly collected from each group, and the ultrastructure of zebrafish intestinal tissues was observed using a Transmission Electron Microscope (TEM) (Hitachi T7700, Tokyo, Japan) (Mhalhel et al. 2023).

### 2.8 Transcriptome Sequencing

RNA‐sequencing analysis was performed on the wild‐type zebrafish larvae samples from the control group, the model group (50 μg/mL TNBS), and the high‐dose GTSFTTTAER group (100 μM GTSFTTTAER). Total RNA was extracted from each sample. After quality control, the library was constructed and sequenced. The transcriptome sequencing analysis was conducted with the assistance of OE Biotech Co. Ltd. (Shanghai, China). Differential expression between two conditions/groups (two biological replicates per condition) was analyzed using the DESeq2 R package (1.20.0). Genes with an adjusted *p*‐value ≤ 0.05 and an absolute fold change ≥ 2 identified by DESeq2 were designated as differentially expressed genes (DEGs). The clusterProfiler R package was used to perform Gene Ontology (GO) enrichment analysis and to examine the statistical enrichment of DEGs in Kyoto Encyclopedia of Genes and Genomes (KEGG) pathways (Lu et al. 2023).

### 2.9 RNA Extraction and RT‐qPCR

The treated zebrafish tissues were collected, and total RNA was extracted from zebrafish samples using the SPARKeasy RNA isolation kit according to the manufacturer's instructions. The cDNAs were obtained by reverse transcription of RNA using the SPAPKscript II RT Plus Kit with random primers. The reverse transcription–quantitative polymerase chain reaction (RT‐qPCR) reaction was performed using the NovoStart SYBR qPCR SuperMix plus. The RT‐qPCR amplification reaction conditions were: 95°C for 60 s, 40 cycles of 95°C for 20 s, 55°C for 30 s, and 72°C for 30 s. Relevant primers were designed for zebrafish genes (Table S1). The standard control gene was *β‐actin*, and the data were processed using the 2^−ΔΔCT^ method to calculate changes in mRNA expression relative to *β‐actin*.

### 2.10 Molecular Docking Analysis

According to the amino acid sequence of peptide GTSFTTTAER, the three‐dimensional structure was obtained by ChemDraw software, which was used as the ligand. The structures of key proteins in the Toll‐like receptor (TLR) signaling pathway and necroptosis signaling pathway were obtained as receptors from UniProt and PDB databases. The Discovery Studio software was used to pretreat the ligand and the receptor. CDOCKER module docked peptide GTSFTTTAER with core protein files TLR3 (1ZIW), TLR4 (4G8A), and RIPK1 (7YDX) to obtain docking results, and CDOCKER‐ENERGY was used to evaluate the binding ENERGY (Zhang et al. 2018).

### 2.11 Statistical Analysis

All the results were expressed as the mean ± standard error of the mean, and one‐way analysis of variance was used to determine significant differences between groups. The statistical significance was set as: ^#^ *p* < 0.05, ^##^ *p* < 0.01 versus the control group; and *p < 0.05, **p < 0.01 versus the TNBS group.

## 3 Results

### 3.1 GTSFTTTAER Inhibited Increase Intestinal Immune Cells in TNBS‐Induced Zebrafish

**FIGURE 2:**
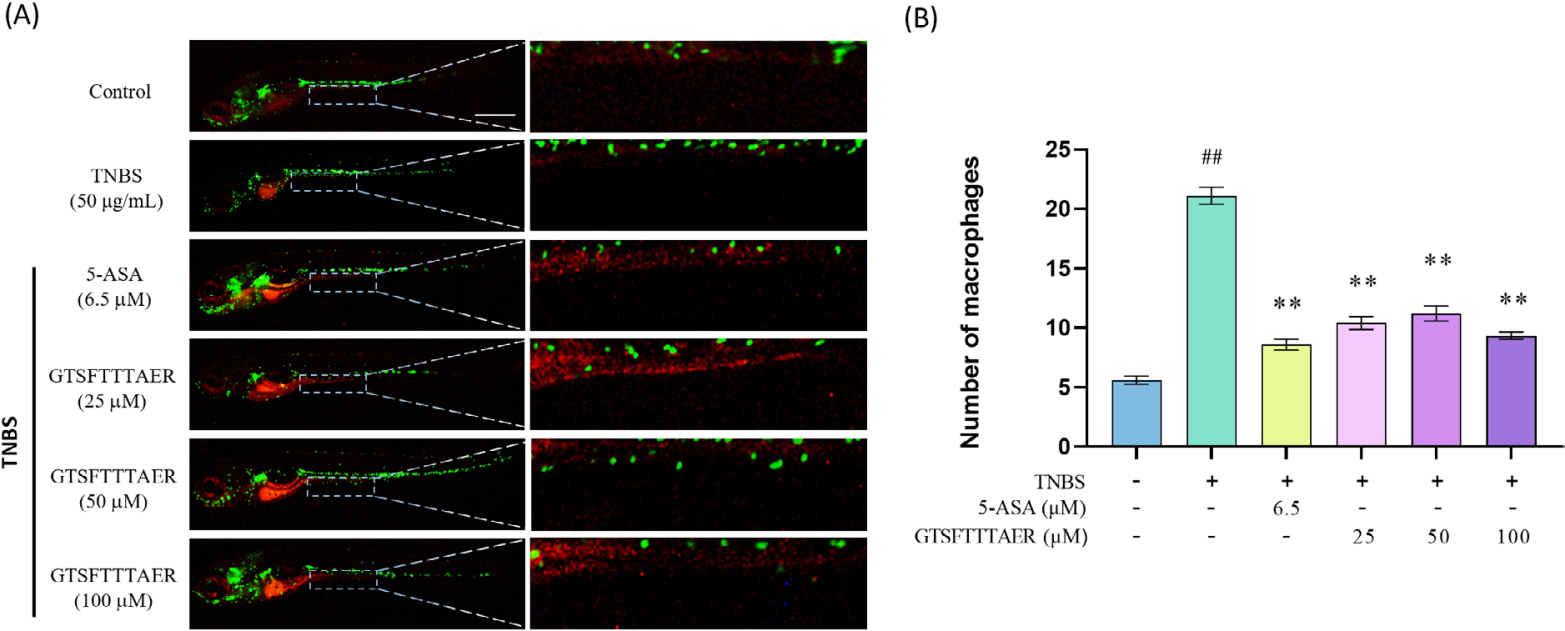
GTSFTTTAER inhibited the 2,4,6‐trinitrobenzene sulfonic acid (TNBS)‐induced increase of immune cells in the zebrafish larvae intestine. Scale bar is 500 μm. (A) Representative fluorescence images of Tg (*zlyz*: EGFP) zebrafish intestine. (B) Statistical analysis of the number of immune cells in the zebrafish intestine. ^##^ *p* < 0.01 versus the control group; **p < 0.01 versus the TNBS group.

To assess the protective effect of the peptide GTSFTTTAER on TNBS‐induced IBD zebrafish, we evaluated the number of immune cells in the zebrafish intestine, an indicator of the severity of intestinal inflammation. Before formal experiments, a series of concentration gradients of the peptide GTSFTTTAER (12.5, 25, 50, 100, 200, 400, 800, and 1600 μM) were set up to determine the optimal experimental concentration. Among these, all zebrafish died at 1600 μM GTSFTTTAER, and dosages below 400 μM were considered to be safe (Figure S1A). The 100 μM GTSFTTTAER had an anti‐inflammatory effect and was a relatively lower dosage compared to other dosages (Figure S1B,C), so it was selected in the following experiments. According to the results in Figure 2, zebrafish in the TNBS group showed a significant increase in intestinal immune cell numbers compared with the control group, indicating that TNBS treatment successfully induced IBD. However, treatment with GTSFTTTAER at different doses (25, 50, and 100 μM) as well as the 5‐ASA significantly alleviated TNBS‐induced inflammation in the zebrafish intestine. The therapeutic efficacy in the 100 μM GTSFTTTAER group was similar to the 5‐ASA positive control group.

### 3.2 GTSFTTTAER Restored TNBS‐Induced Impairment in Gastrointestinal Motility of Zebrafish

**FIGURE 3:**
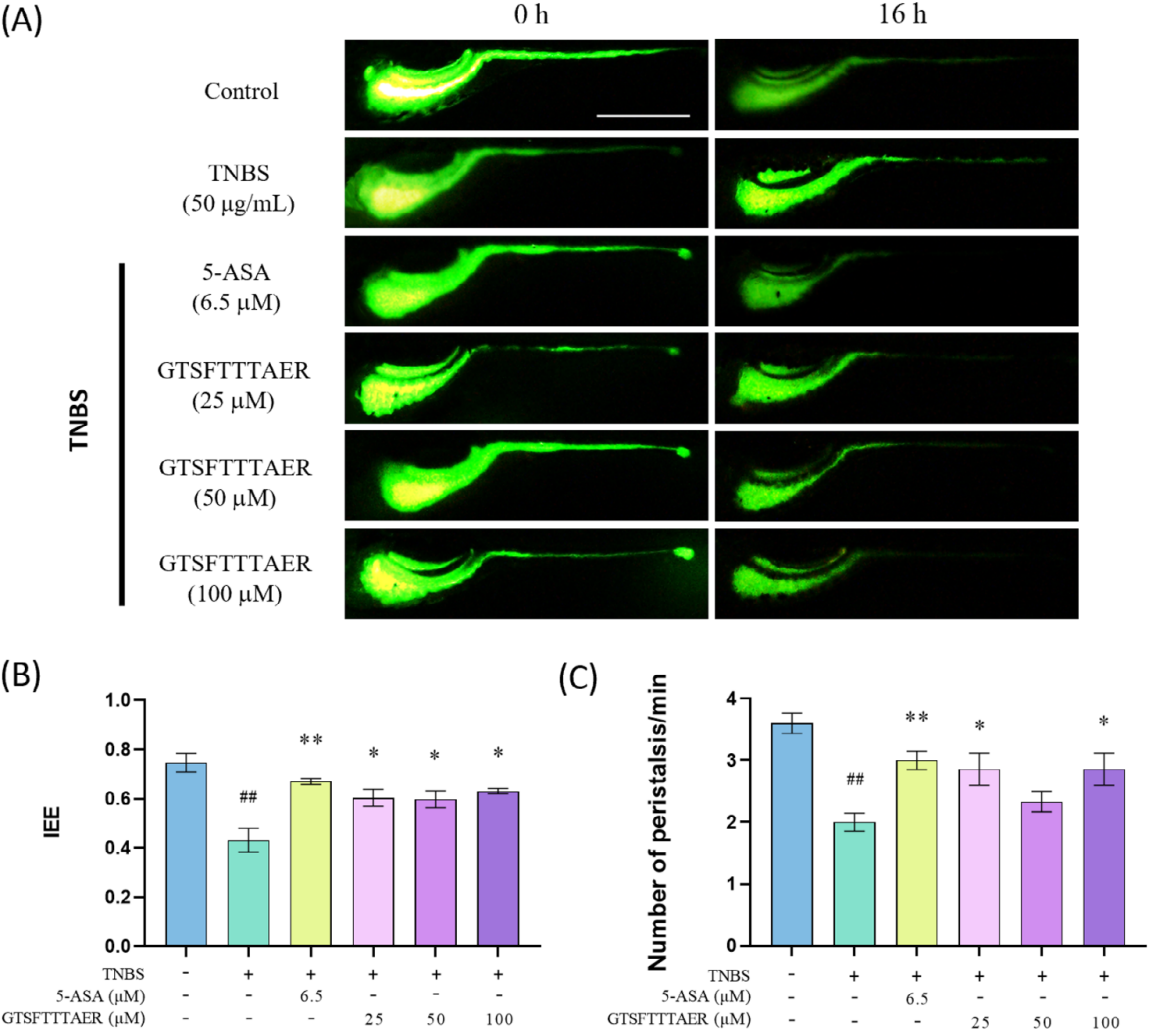
Improvement of GTSFTTTAER on TNBS‐induced intestinal motility impairment of zebrafish larvae. Scale bar is 500 μm. (A) Typical fluorescence images of wild‐type zebrafish intestine. (B) Statistical analysis of the intestinal efflux efficiency. (C) Statistical analysis of the frequency of intestinal peristalsis. ^##^ *p* < 0.01 versus the control group; *p < 0.05, **p < 0.01 versus the TNBS group.

In this experiment, zebrafish were directly immersed in a calcein solution to observe the effect of GTSFTTTAER on IBD. Compared with the blank control group, the IOD of the intestinal tract of zebrafish larvae in the TNBS group was significantly increased, and the IEE and peristalsis times were also significantly decreased. Compared with the TNBS group, zebrafish in the 5‐ASA group and GTSFTTTAER group showed significantly higher IEE and a quicker frequency of peristalsis (Figure 3). This indicates that the peptide GTSFTTTAER could effectively restore both the IEE and peristaltic frequency in zebrafish with IBD.

### 3.3 Effects of GTSFTTTAER on TNBS‐Induced Intestinal Tissue

**FIGURE 4:**
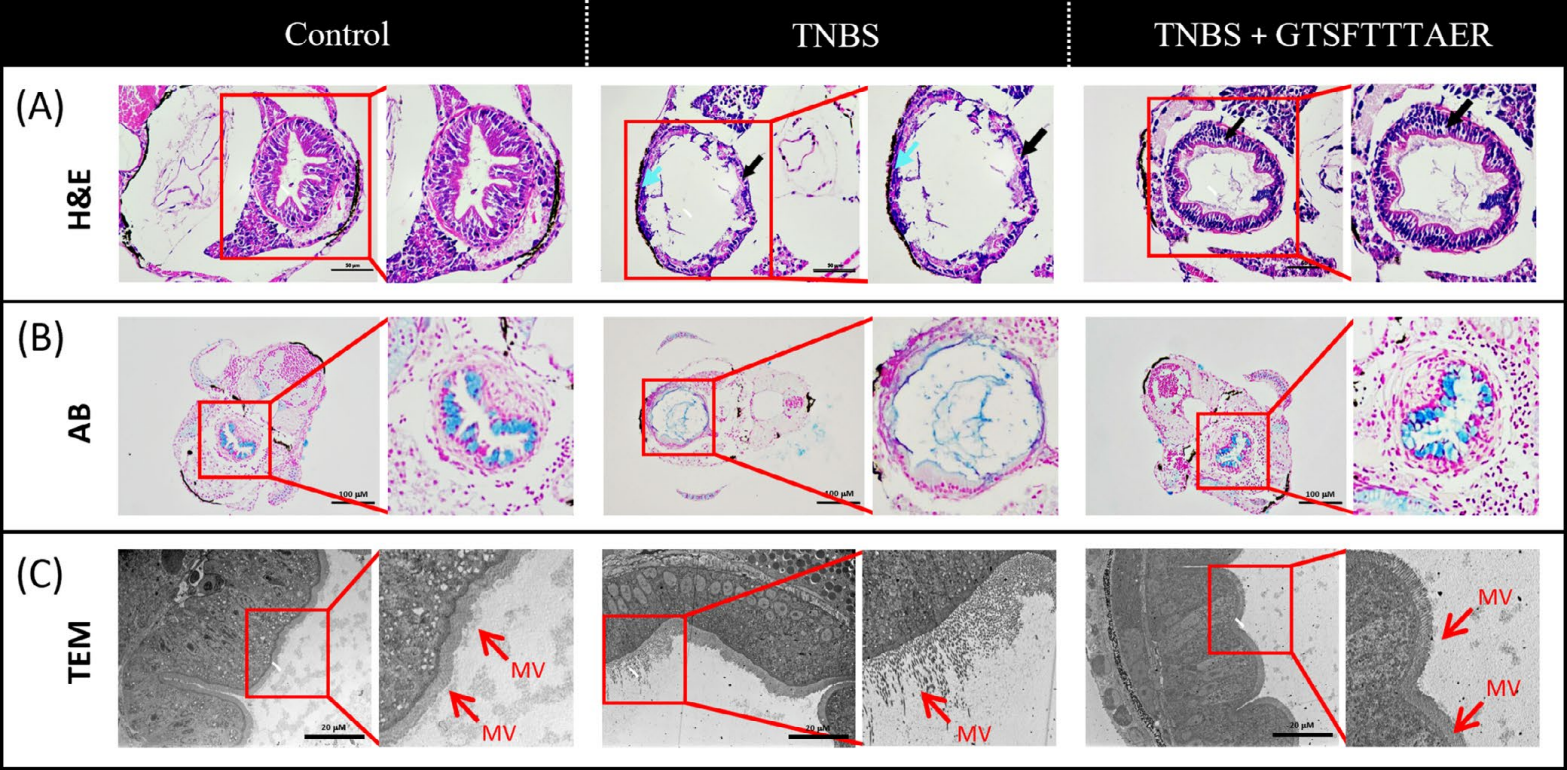
Effects of GTSFTTTAER on TNBS‐induced intestinal tissue of zebrafish larvae. (A) Hematoxylin and Eosin staining of intestinal tissues. Black arrow: The state of intestinal cells; blue arrow: Mucosal layer necrosis, cell lysis, disappearance of intestinal folds. Scale bar is 50 μm. (B) Alcian blue staining of intestinal tissues. Scale bar is 100 μm. (C) Transmission electron microscopy of the intestinal ultrastructure in zebrafish. Mv represents intestinal microvilli. Scale bar is 20 μm.

The results of H&E staining of zebrafish intestinal tissues are presented in Figure 4A. In the control group, the structure of the mucosa, muscularis propria, and serosa was distinctly visible, with no significant abnormalities observed in any of the layers. The mucosal surface was lined with a single layer of well‐arranged columnar epithelial cells, while the muscularis propria comprised connective tissue with uniformly stained and regularly arranged myocytes. The serosa layer was notably thin. Compared with the control group, the intestine of zebrafish in the TNBS group showed sparse cell arrangement (black arrow), necrosis of the mucosal layer, cell lysis, enhanced cytoplasmic basophilia, and the disappearance of intestinal folds (blue arrow). However, the intestine of zebrafish in the GTSFTTTAER group showed a clear structure of all layers. This group demonstrated recovery of intestinal folds, tightly arranged cells, no obvious cell necrosis, and a few instances of watery degeneration (black arrow).

In the control group, AB staining revealed a well‐defined mucus layer, while the TNBS group showed a significant reduction in mucus thickness. It has been indicated that the zebrafish intestinal lumen has reduced acidic proteins, along with diminished secretory function of intestinal goblet cells in the TNBS group (Jia et al. 2024). In comparison, the GTSFTTTAER group significantly increased the thickness of the mucus layer and improved secretion of goblet cells in TNBS‐induced zebrafish (Figure 4B).

As shown by the TEM results, zebrafish intestinal microvilli in the control group were abundant, neatly arranged, and uniform in length. Compared with the control group, zebrafish intestinal microvilli in the TNBS group were slightly fewer in number, sparsely arranged locally, and uneven in thickness. Notably, compared with the TNBS group, the length and number of intestinal microvilli were significantly restored in the GTSFTTTAER treatment group (Figure 4C).

### 3.4 Transcriptomic Analysis of GTSFTTTAER to Ameliorate TNBS‐Induced IBD Injury

**FIGURE 5:**
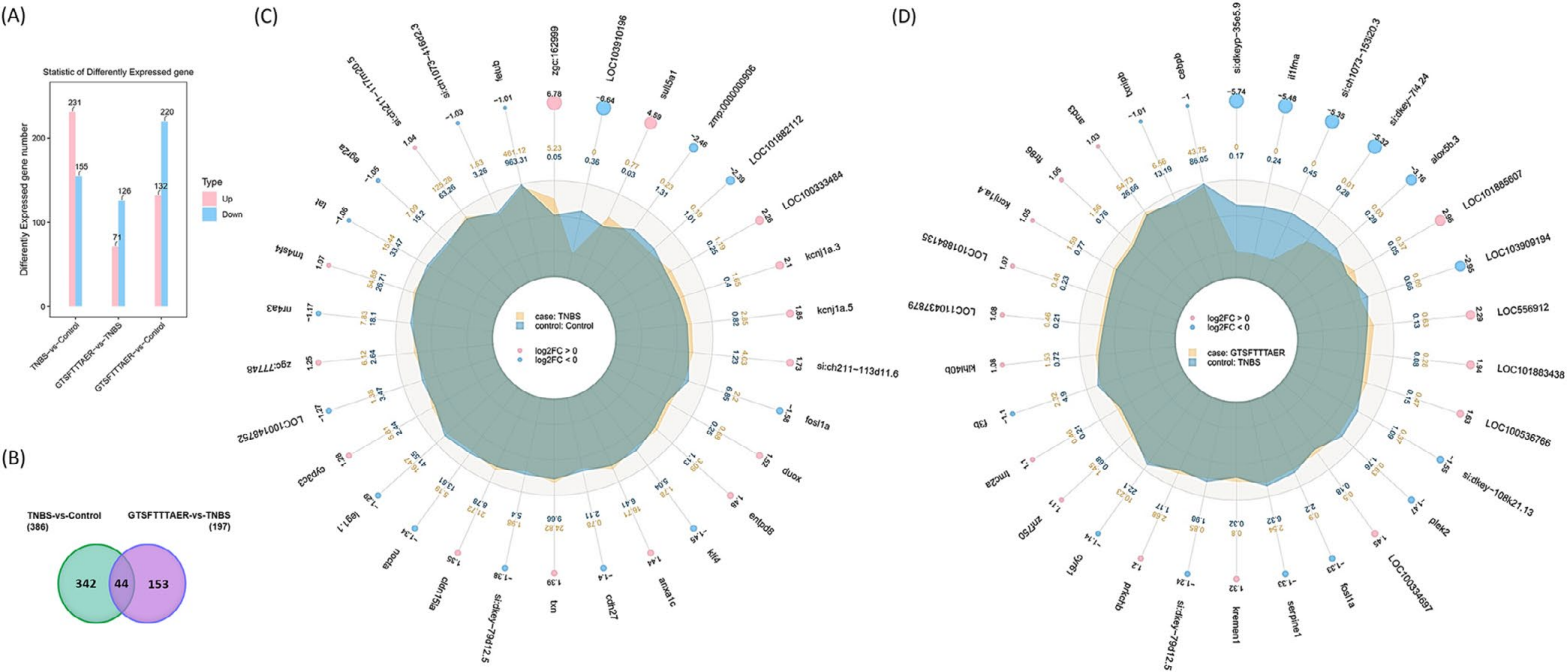
Analysis of differentially expressed genes (DEGs). (A) Histogram of DEGs statistics for TNBS versus Control, GTSFTTTAER versus TNBS, and GTSFTTTAER versus Control. (B) Venn diagram of common and unique DEGs between the TNBS versus Control and GTSFTTTAER versus TNBS comparison groups. (C) Differential gene radar chart for TNBS versus Control. (D) Differential gene radar chart for GTSFTTTAER versus TNBS. First circle: Up‐regulated genes are light red and down‐regulated genes are light blue; the size of the circle varies according to the size of the log2(FC) value. Second circle: The data represent the average expression for each gene in groups.

The peptide GTSFTTTAER has been shown to phenotypically relieve symptoms in zebrafish with IBD, but its mechanism of action remains unclear. In order to explore the mechanism of GTSFTTTAER, we performed an analysis of zebrafish genes in the control group, the TNBS group, and the high‐dose GTSFTTTAER group. As can be easily seen in Figure 5A. A total of 386 DEGs were identified between the TNBS group and the control group; of these, 231 genes were up‐regulated and 155 genes were down‐regulated. Compared with the TNBS group, 197 DEGs were found in the GTSFTTTAER group, with 71 genes up‐regulated and 126 genes down‐regulated. Furthermore, Venn analysis showed there were 44 common DEGs in two comparison groups: TNBS versus Control and GTSFTTTAER versus TNBS (Figure 5B). Among the DEGs, TLR4 and RIPK1 were significantly downregulated in the GTSFTTTAER group. TLR4 is a receptor for bacterial LPS and regulates the LPS‐induced inflammatory response (Kim et al. 2020). Similarly, RIPK1 binds to the major necroptotic regulator RIPK3 to form a necroptotic complex, leading to intestinal epithelial cell death and barrier dysfunction (Li, Xu, et al. 2022). The details of the relatively up‐ and down‐regulated DEGs can be shown in Figure 5C,D.

#### 3.4.1 GO Enrichment Analysis of DEGs

**FIGURE 6:**
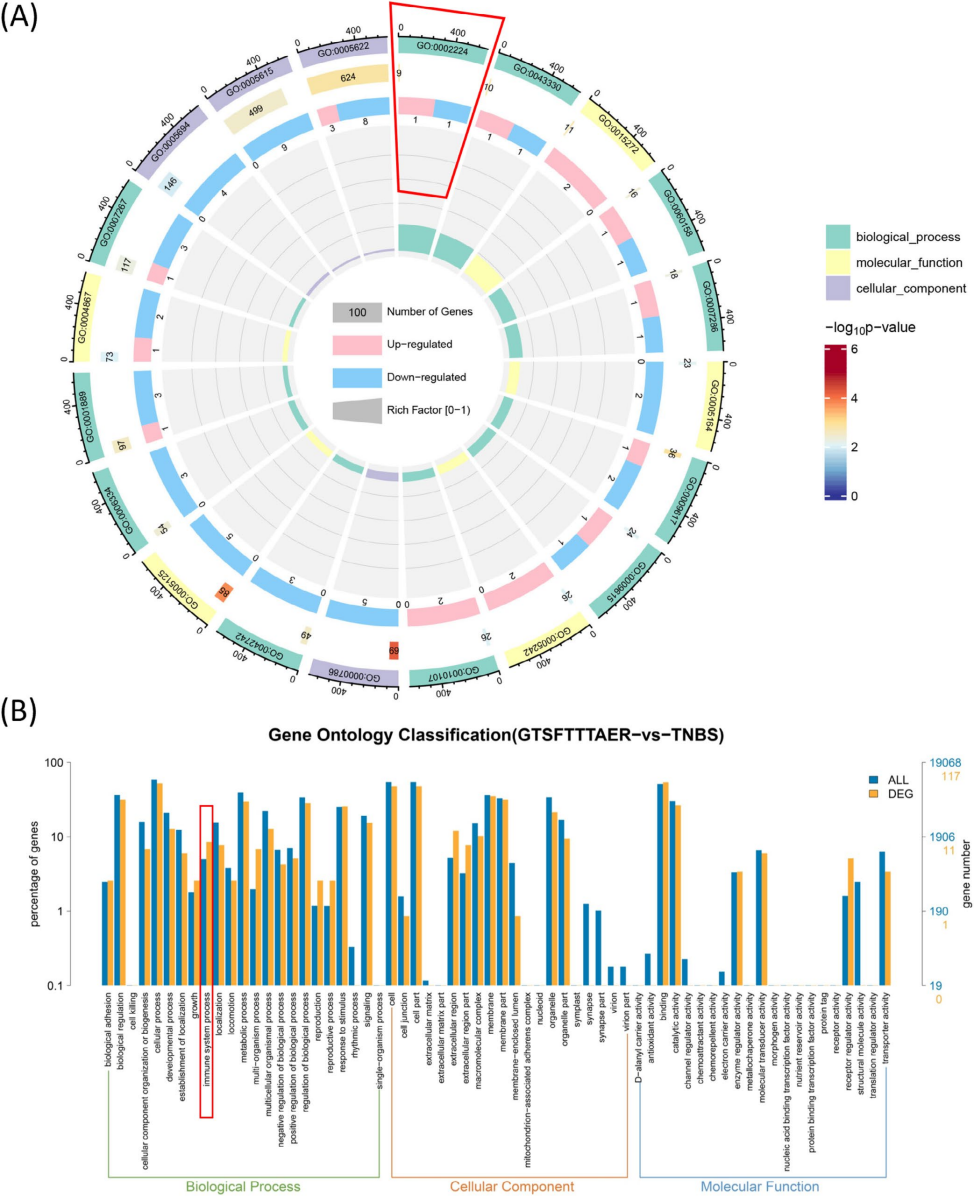
Gene Ontology (GO) enrichment analysis of DEGs in the GTSFTTTAER versus TNBS group. (A) Circle plot of DEGs and all genes in GO enrichment analysis. (B) Comparative plot of the distribution of DEGs and all genes at GO Level 2.

Next, we conducted GO enrichment analysis on the DEGs from the GTSFTTTAER versus TNBS comparison group to explore changes in functional gene expression. As shown in the circular graph (Figure 6), both up‐regulated and down‐regulated genes in the GTSFTTTAER versus TNBS group were most significantly enriched in the TLR signaling pathway (GO: 0002224), which is associated with immune system processes—a key category in the biological process GO analysis. The GO terms and the distribution of DEGs at GO Level 2 are in Tables S2 and S3.

#### 3.4.2 KEGG Enrichment Analysis of DEGs

**FIGURE 7:**
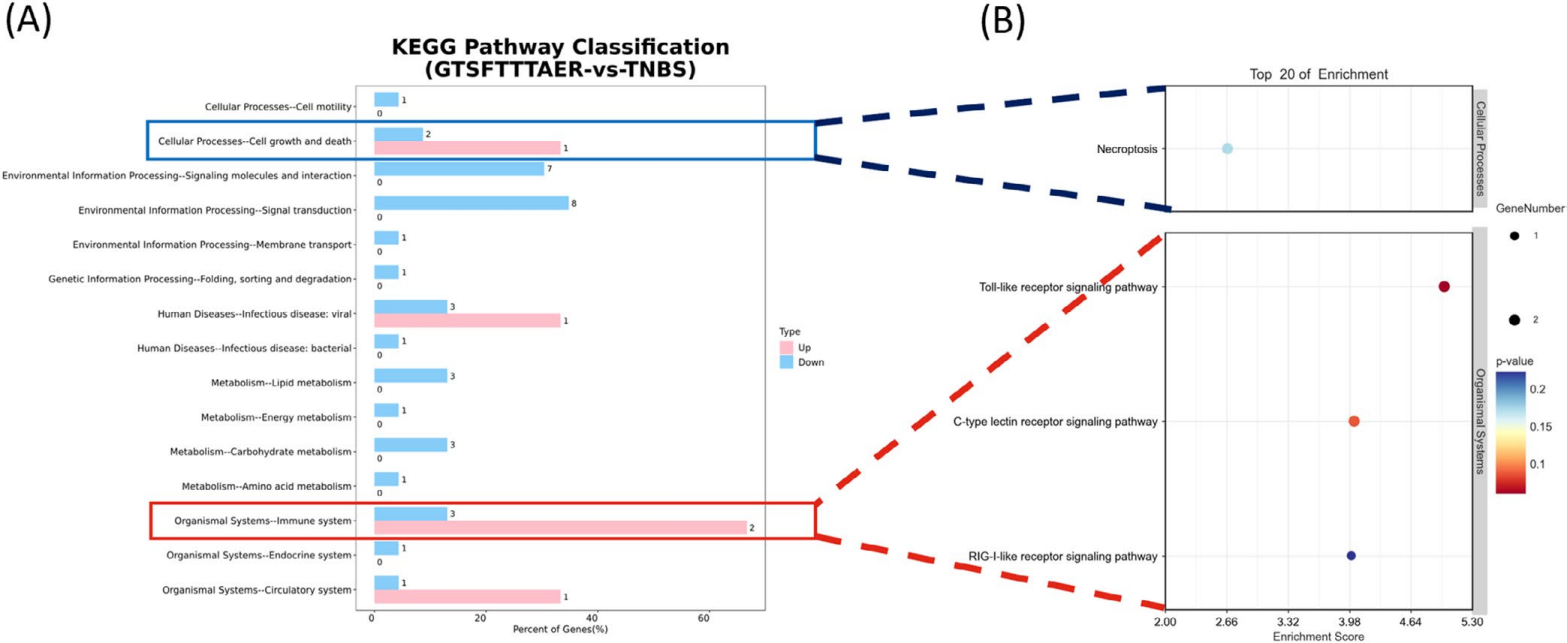
Kyoto Encyclopedia of Genes and Genomes (KEGG) analysis of DEGs in the GTSFTTTAER versus TNBS group. (A) KEGG Level2 distribution map of up‐regulated and down‐regulated DEGs. (B) Bubble diagram of DEGs in the KEGG enrichment analysis.

In KEGG analysis, up‐ and down‐regulated genes in the GTSFTTTAER‐versus‐TNBS comparison were mainly distributed in Cellular Processes‐Cell growth and death, Environmental Information Processing‐Signaling molecules and interaction, Environmental Information Processing‐Signal transduction, Human Diseases‐Infectious disease: viral, and Organismal Systems‐Immune system, etc. The detailed distribution of up‐ and down‐regulated DEGs is presented in Table S4. In particular, the genes of Organismal Systems‐Immune system were most significantly up‐regulated (Figure 7A). Among these, the TLR signaling pathway and necroptosis signaling pathway were significantly enriched, too (Figure 7B). This indicates that the peptide GTSFTTTAER may exert its therapeutic effects in IBD zebrafish through modulation of the TLR and necroptosis signaling pathway.

### 3.5 GTSFTTTAER Attenuates IBD Injury Through TLR Signaling Pathway and Necroptosis Pathway

**FIGURE 8:**
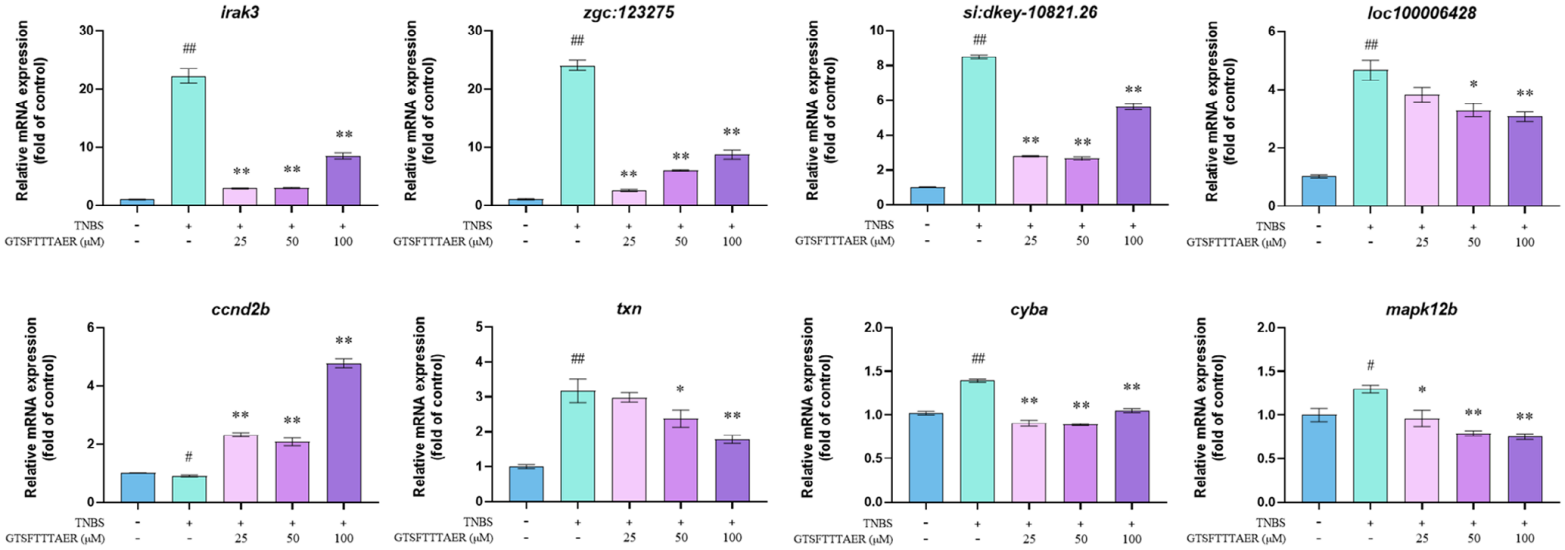
Reverse transcription‐quantitative polymerase chain reaction results of peptide GTSFTTTAER on the expression of representative genes screened by transcriptome. ^#^ *p* < 0.05, ^##^ *p* < 0.01 versus the control group; *p < 0.05, **p < 0.01 versus the TNBS group.

The KEGG analysis showed that the DEGs were mainly enriched in 15 pathways, of which the TLR and necroptosis signaling pathways were the most relevant to IBD. To validate the RNA sequencing results, we conducted RT‐qPCR on eight differentially expressed genes. The peptide GTSFTTTAER can down‐regulate *irak3*, *zgc:123275*, *si:dkey‐10821.26*, *loc100006428*, *txn*, *cyba*, *mapk12b*, and up‐regulate *ccnd2b* in TNBS‐induced zebrafish. These results demonstrated that the transcript levels of validated genes from the transcriptome were consistent with the transcriptome sequencing results (Figure 8).

**FIGURE 9:**
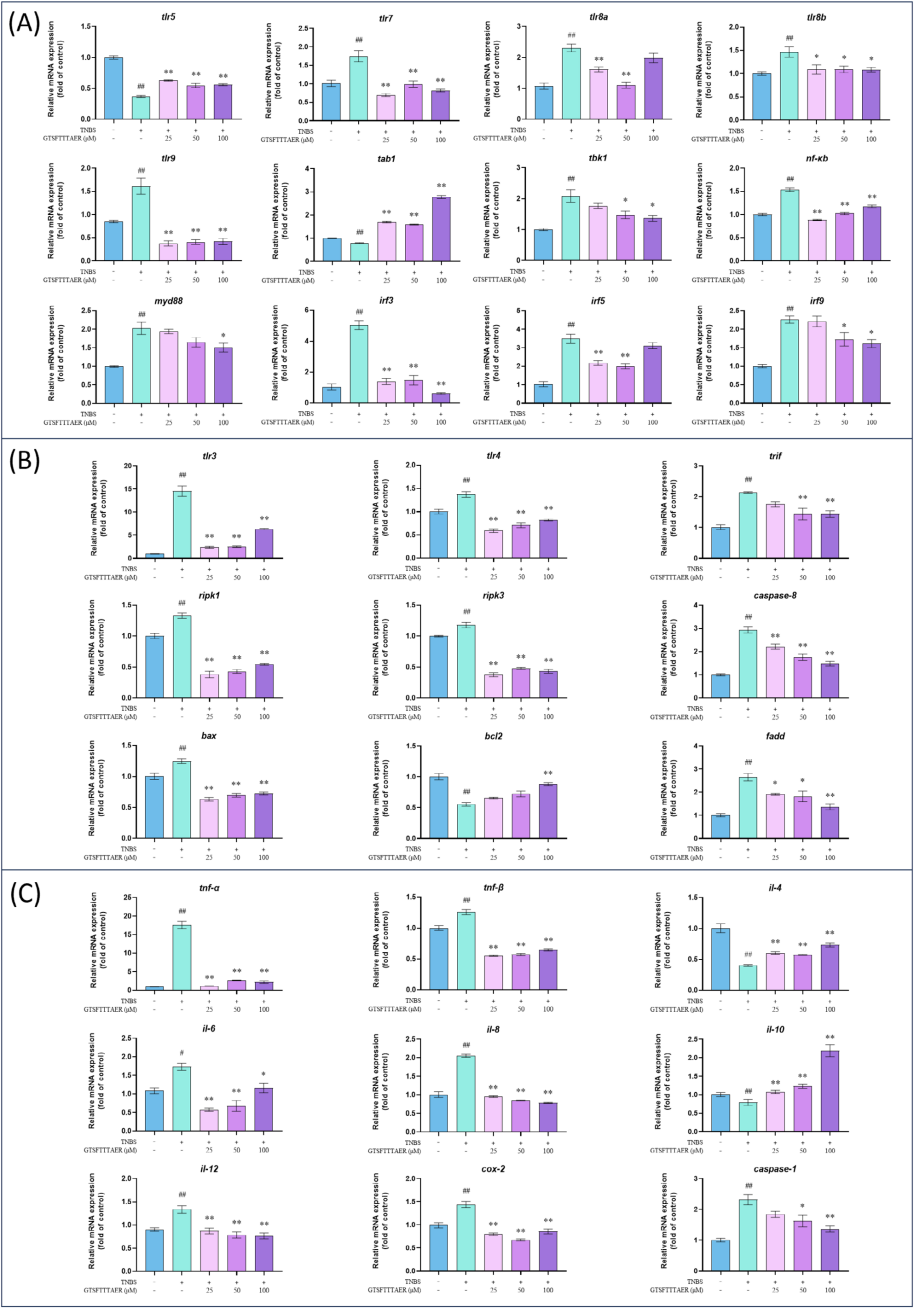
Effect of peptide GTSFTTTAER at the RNA level on inflammatory bowel disease in zebrafish. (A) Expression of genes related to Toll‐like receptor (TLR) signaling pathway. (B) Expression of genes related to the necroptosis signaling pathway. (C) Expression of inflammation‐related genes. ^#^ *p* < 0.05, ^##^ *p* < 0.01 versus the control group; *p < 0.05, **p < 0.01 versus the TNBS group.

We propose that the TLR signaling pathway and necroptosis signaling pathway in the KEGG analysis are essential for the ameliorative effects of the peptide GTSFTTTAER on IBD. As a consequence, we quantified the DEGs related to these signaling pathways in the control group, the TNBS group, and the peptide GTSFTTTAER group. Compared with the control group, the TNBS group showed significant changes in gene expression. Specifically, the gene expression levels of *tlr3*, *tlr4*, *tlr7*, *tlr8a*, *tlr8b*, *tlr9*, *trif*, *tnf‐α*, *tnf‐β*, *tbk1*, *nf‐κb*, *myd88*, *irf3*, *irf5*, *irf9*, *ripk1*, *ripk3*, *caspase‐1*, *caspase‐8*, *cox‐2*, *fadd*, *bax*, *il‐6, il‐8*, and *il‐12* were significantly up‐regulated; while *tlr5*, *tab1*, *bcl2*, *il‐4*, and *il‐10* were significantly down‐regulated. However, TNBS‐regulated gene expression levels were significantly reversed by treatment with the peptide GTSFTTTAER (Figure 9).

### 3.6 Molecular Docking Results

**FIGURE 10:**
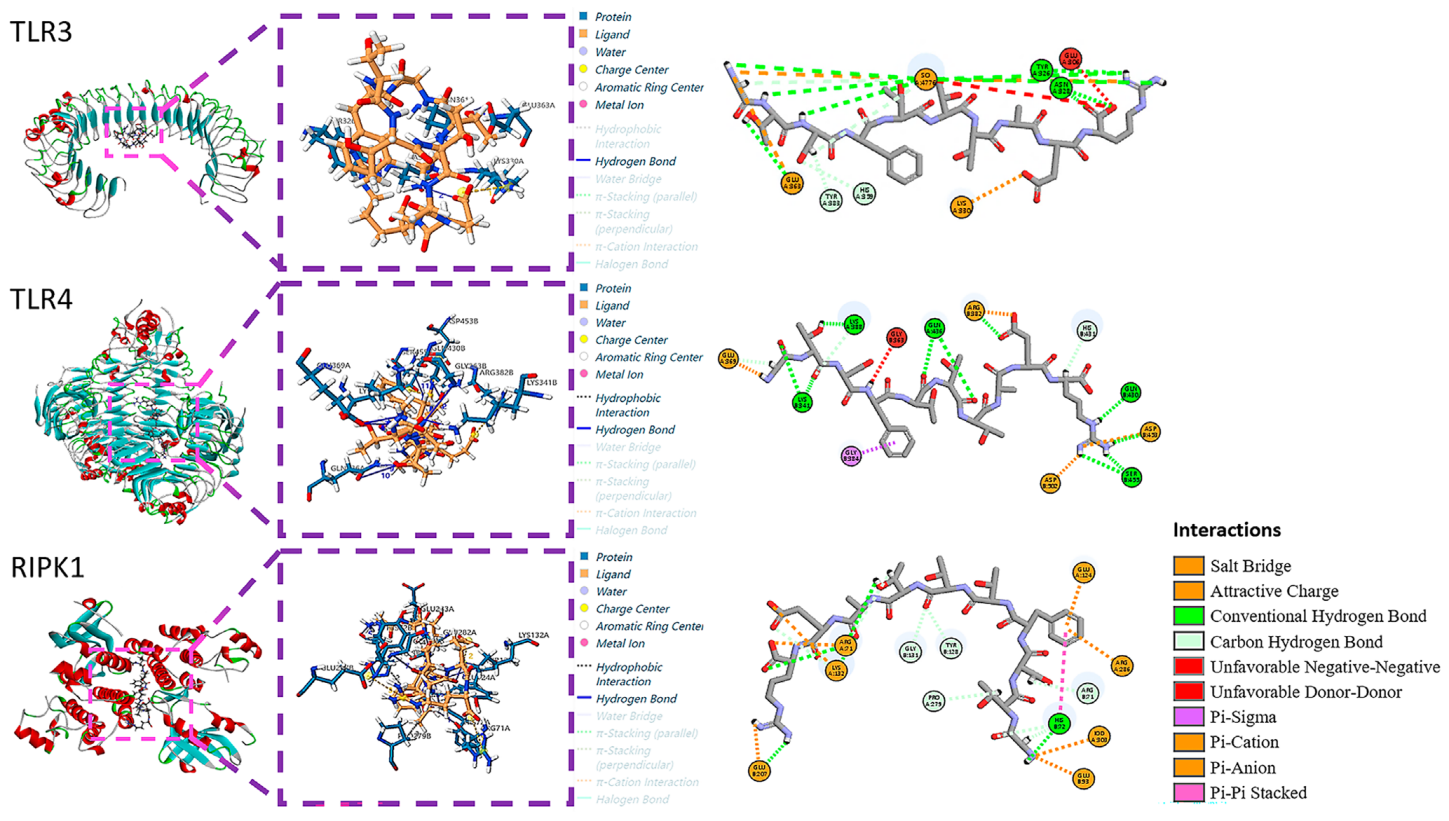
The molecular docking of peptide GTSFTTTAER to TLR3, TLR4, and RIPK1 protein.

In order to predict the binding affinity of the peptide GTSFTTTAER to its targets, we used the peptide as a ligand and selected key target proteins TLR3 (1ZIW), TLR4 (4G8A), and RIPK1 (7YDX) from the TLR signaling pathway and necroptosis signaling pathway as receptors for docking. As shown in Figure 10, the peptide GTSFTTTAER binds to these receptors through hydrogen bonding, electrostatic force, and hydrophobic force interaction. Respectively, the binding energies of GTSFTTTAER to TLR3, TLR4, and RIPK1 were −135.478, −132.927, and −156.399 kJ/mol. These CDOCKER‐ENERGY results indicate that the peptide GTSFTTTAER exhibits a high affinity for TLR3, TLR4, and RIPK1.

## 4 Discussion

Inflammatory bowel disease is a chronic, recurrent condition closely associated with immune dysfunction (Ye and Lai 2021). With the change of people's lifestyle and the increasing pressure of life, the global incidence of IBD is increasing year by year. However, the pathogenesis of IBD is still in the exploratory stage, and existing therapeutic drugs all have different side effects, so there is a broad demand for developing a new drug. In particular, organisms are under close scrutiny in the resource‐rich deep sea. Peptides represent a unique class with a wide range of biological activities and enhanced efficacy, triggering concern in exploring treatments for IBD. Compared to larger biomolecules, the peptide GTSFTTTAER has a relatively deeper tissue penetration due to its smaller size. The metabolites of peptides are usually amino acids with short half‐lives and low immunogenicity. At the same time, these inherent properties of peptide drugs determine limitations such as weaker stability and potential toxicity (Bucataru and Ciobanasu 2024). However, it is possible to modulate the pharmacokinetic characteristics of the peptide by synthesizing peptidomimetics or using structural modifications, and thus better overcome the limitations (Sharma et al. 2023).

Patients with IBD commonly experience symptoms such as digestive function abating, gut swelling, intestinal peristalsis slowing, and intestinal injury. The phenomena of TNBS‐induced macrophage aggregation and reduced peristalsis in the intestinal area of zebrafish are very similar to the clinical symptoms of IBD (Pan et al. 2022; Xu et al. 2024). In our study, we observed that low (25 μM), medium (50 μM), and high (100 μM) dosages of GTSFTTTAER inhibited the aggregation of immune cells in the intestinal area of zebrafish induced by TNBS. In addition, the peptide GTSFTTTAER showed significant improvement in IEE and intestinal peristalsis frequency in zebrafish with IBD. Furthermore, we also evaluated the activity of other peptides such as MVLLGVLMG. The results showed that the MVLLGVLMG groups had no anti‐inflammatory activity at low, medium, and high concentrations (Figure S2). These results suggest that the peptide GTSFTTTAER can alleviate the intestinal inflammation of zebrafish and has a therapeutic effect on IBD.

Apart from the inflammatory phenotypic damage, IBD model zebrafish also show other intestinal tissue lesions (Uyttebroek et al. 2020). Histological results show inflammatory infiltration, goblet cells' secretion dysfunction, and intestinal microvilli defects in IBD zebrafish. Notably, high‐dose peptide (100 μM) significantly ameliorated TNBS‐induced intestinal tissue dysfunction in zebrafish, as demonstrated by the H&E staining, AB staining, and TEM results.

To further elucidate the underlying mechanisms, we conducted transcriptome analysis of zebrafish from the blank control group, TNBS‐induced model group, and the group treated with high‐dose peptide GTSFTTTAER (100 μM). The results of GO and KEGG enrichment analysis revealed that the DEGs from the high‐dosage peptide GTSFTTTAER group and the TNBS group were mainly enriched in the immune system‐related TLR signaling pathway and necroptosis signaling pathway.

As we all know, the intestinal immune system plays a vital role in maintaining the balance of intestinal microbiota, which is closely related to the pathophysiology of IBD (Saez et al. 2023). TLRs serve as central regulators of immune system development and function, with TLR‐related gene expression directly influencing immune‐mediated microbiota regulation (Aluri et al. 2021). Our RT‐qPCR results showed that TLR signaling‐related genes (*tlr3*, *tlr4*, *tlr5*, *tlr7*, *tlr8a*, *tlr8b*, *tlr9*, *tab1*, *tbk1*, *nf‐κb*, *myd88*, *irf3*, *irf5*, *irf9*), necroptosis signaling‐related genes (*tlr3*, *tlr4*, *trif*, *ripk1*, *ripk3*, *caspase‐8*, *fadd*, *bax*, *bcl2*), and inflammatory factors (*tnf‐α*, *tnf‐β*, *il‐4*, *il‐6*, *il‐8*, *il‐10, il‐12*, *caspase‐1, cox‐2*) were aberrantly expressed in zebrafish with IBD. GTSFTTTAER treatment normalized the dysregulated expression of these genes, demonstrating its dual regulatory capacity across both TLR and necroptosis pathways. In the TLR family members, TLR1, TLR2, TLR4, and TLR5 are receptors expressed on the cell surface, while TLR3, TLR7, TLR8, and TLR9 are receptors expressed on the endosomal membrane of the cell (Luchner et al. 2021). These TLRs, located at different positions in the cell, also play important roles in the anti‐inflammatory immune response. Of these, TLR3, TLR4, TLR5, TLR7, TLR8, and TLR9 have been shown to exert an anti‐inflammatory immune role by regulating the expression of inflammatory factors and interferon regulatory factors (IRFs), such as IRF3, IRF5, IRF7, and IRF9 (Heinz et al. 2020; Hu et al. 2021; Ying et al. 2019). TLR activation (TLR3/4) triggers downstream signaling cascades involving MyD88 and TRIF, which not only drive NF‐κB‐mediated inflammation but also intersect with necroptosis regulators such as RIPK1 and RIPK3 (Ciesielska et al. 2021; Ryu et al. 2022). TLR pathway components interface with necroptosis execution mechanisms, creating a pathogenic signaling nexus in IBD (Schünke et al. 2021; Yu et al. 2021).

The transcriptomic and RT‐qPCR data show that in the TNBS‐induced IBD model, the expression of TLR3, TLR4, and RIPK1 is up‐regulated, which is consistent with previous studies. Studies have shown that TLR3 may be involved in intestinal epithelial mucosal injury (Zhou et al. 2023). TLR4 participates in both in vitro and in vivo inflammatory responses and can ameliorate intestinal damage in experimental animals with IBD (Cao et al. 2022). In addition, RIPK1 can alleviate multiorgan inflammation through the regulation of necroptotic activation (Chiou et al. 2024). TLR4 signaling via the TRIF‐dependent pathway recruits RIPK1 to form a necrosome complex, linking inflammatory signaling to necroptotic cell death (Ahn and Prince 2017; Hernandez et al. 2017; Hu et al. 2016). Given the important roles of TLR3, TLR4, and RIPK1 in the pathophysiology of IBD, we further explored the potential interactions between peptide GTSFTTTAER and target proteins through molecular docking. The results revealed that the peptide GTSFTTTAER has a strong binding capacity with RIPK1, potentially interfering with its interaction with TRIF or other components of the necrosome. This blocking effect can simultaneously weaken both the NF‐κB‐mediated inflammatory response and RIPK1/RIPK3‐dependent necroptosis. The downregulation of inflammatory factors and necroptosis markers after treatment supports this mechanism.

## 5 Conclusions

In summary, the peptide GTSFTTTAER shows a potential role in the treatment of IBD. We found that TNBS‐induced zebrafish larvae exhibited increased macrophage numbers, decreased IEE, and peristalsis frequency. Additionally, there was damage to the intestinal tissue and a reduction in goblet cell secretion. Meanwhile, the peptide GTSFTTTAER ameliorated these inflammatory responses in IBD. On the basis of the transcriptome analysis, RT‐qPCR, and molecular docking results, the ability of peptide GTSFTTTAER to treat IBD may be associated with the TLR signaling pathway and necroptosis pathway. This article elucidates the anti‐IBD mechanism of peptide GTSFTTTAER extracted from *Rapana venosa*. We believe that our findings will provide a solid experimental basis for the further development of peptide GTSFTTTAER as a potential therapeutic agent against IBD.

## Author Contributions

**Qifei Wang:** data curation (equal), investigation (equal), visualization (equal), writing – original draft (equal), writing – review and editing (equal). **Fenghua Xu:** data curation (equal), investigation (equal), visualization (equal), writing – original draft (equal), writing – review and editing (equal). **Yongna Cao:** data curation (equal), formal analysis (lead), investigation (equal), methodology (lead), writing – original draft (equal). **Bin Li:** data curation (supporting), investigation (supporting), supervision (lead), validation (lead). **Shanshan Zhang:** conceptualization (lead), formal analysis (supporting), methodology (equal). **Yun Zhang:** funding acquisition (lead), methodology (equal), project administration (lead).

## Disclosure

All claims expressed in this article are solely those of the authors and do not necessarily represent those of their affiliated organizations, or those of the publisher, the editors, and the reviewers. Any product that may be evaluated in this article, or claim that may be made by its manufacturer, is not guaranteed or endorsed by the publisher.

## Ethics Statement

All animal experiments were performed in accordance with the Standard Ethical Guidelines of the Committee of the Biology Institute, Qilu University of Technology (Shandong Academy of Sciences) (Approval No. SWS20210225).

## Conflicts of Interest

The authors declare no conflicts of interest.

## Supporting information


**Table S1.** Primer sequence.
**Table S2.** Terms of GOids in GO enrichment analysis circle plot.
**Table S3.** Comparative table of the distribution of DEGs and all genes at GO Level 2.
**Table S4.** Distribution of up‐ and down‐regulated DEGs of GTSFTTTAER versus TNBS at KEGG Level 2.
**Figure S1.** Effects of different concentrations of peptide GTSFTTTAER in zebrafish. (A) Morphological diagram of the zebrafish. Scale bar is 1 mm. (B) Fluorescent immune cell images in Tg (*zlyz*: EGFP) zebrafish intestine. Scale bar is 500 μm. (C) Statistical analysis of the number of immune cells in the zebrafish intestine. ^##^ *p* < 0.01 versus the control group; **p < 0.01 versus the TNBS group.
**Figure S2.** Effects of the peptide MVLLGVLMG on TNBS‐induced zebrafish larvae. (A) Fluorescent immune cell images in Tg (*zlyz*: EGFP) zebrafish intestine. Scale bar is 500 μm. (B) Statistical analysis of the number of immune cells in the zebrafish intestine. (C) Representative fluorescence images of wild‐type zebrafish intestine. Scale bar is 500 μm. (D‐E) Statistical analysis of the intestinal efflux efficiency and the frequency of intestinal peristalsis. ^##^ *p* < 0.01 versus the control group; **p < 0.01 versus the TNBS group.

## Data Availability

The data presented in this current study are available from the corresponding author upon reasonable request.
