# Supplementary material for: Peptide GTSFTTTAER From Rapana venosa Alleviates TNBS‐Induced Inflammatory Bowel Disease in a Zebrafish Model via Multi‐Pathway Regulation
Source: Food Sci Nutr. 2025 Jun 12;13(6):e70427. doi: 10.1002/fsn3.70427 (PMC12162357; doi:10.1002/fsn3.70427)
Supplement: Supplementary file 1 — Table S1. Primer sequence. Table S2. Terms of GOids in GO enrichment analysis circle plot. Table S3. Comparative table of the distribution of DEGs and all genes at GO Level 2. Table S4. Distribution of up‐ and down‐regulated DEGs of GTSFTTTAER versus TNBS at KEGG Level 2. Figure S1. Effects of different concentrations of peptide GTSFTTTAER in zebrafish. (A) Morphological diagram of the zebrafish. Scale bar is 1 mm. (B) Fluorescent immune cell images in Tg (zlyz: EGFP) zebrafish intestine. Scale bar is 500 μm. (C) Statistical analysis of the number of immune cells in the zebrafish intestine. ## p < 0.01 versus the control group; **p < 0.01 versus the TNBS group. Figure S2. Effects of the peptide MVLLGVLMG on TNBS‐induced zebrafish larvae. (A) Fluorescent immune cell images in Tg (zlyz: EGFP) zebrafish intestine. Scale bar is 500 μm. (B) Statistical analysis of the number of immune cells in the zebrafish intestine. (C) Representative fluorescence images of wild‐type zebrafish intestine. Scale bar is 500 μm. (D‐E) Statistical analysis of the intestinal efflux efficiency and the frequency of intestinal peristalsis. ## p < 0.01 versus the control group; **p < 0.01 versus the TNBS group. [file FSN3-13-e70427-s001.docx]

**Table S1** Primer sequence.

| **Gene** | **Primer orientation** | **Nucleotide sequence** |
| --- | --- | --- |
| *β-actin* | Forward | 5’-GCATTGCTGACCGTATGCAG-3’ |
|  | Reverse | 5’-ACTCCTGCTTGCTGATCCAC-3’ |
| *irak3* | Forward | 5’-AAGAGCTGATGTGGTCGTGG-3’ |
|  | Reverse | 5’-GCGTGACCCATCTCATCCAG-3’ |
| *zgc:123275* | Forward | 5’-TGAACCGGATGGATTCGTGG-3’ |
|  | Reverse | 5’-TTTCCAGAGCCACACCACAG-3’ |
| *si:dkey-108k21.26* | Forward | 5’-GAAGAGGCAAAACCGGAGGA-3’ |
|  | Reverse | 5’-CCCTTACGGAGAAGCCTGTG-3’ |
| *loc100006428* | Forward | 5’-GCATAAGCTCGCCACTGAGA-3’ |
|  | Reverse | 5’-TGTCAAACAGGTACTCCGCC-3’ |
| *ccnd2b* | Forward | 5’-AGACAGATACGTTCCGCAGG-3’ |
|  | Reverse | 5’-CCACGTCGCAACCATTTTCC-3’ |
| *txn* | Forward | 5’-AGTTGGTGGTGGTGGACTTC-3’ |
|  | Reverse | 5’-CCACATAAAGCGGCCACATC-3’ |
| *cyba* | Forward | 5’-CGAAGATTGAGTGGGCGATG-3’ |
|  | Reverse | 5’-CTCTGAACTGACCAGCCACC-3’ |
| *mapk12b* | Forward | 5’-AGGGCAGGATTTTATCGCCA-3’ |
|  | Reverse | 5’-ACTCGAACTCCTGTCTTGCG-3’ |
| *tlr3* | Forward | 5’-TGAGTTGGAGCATCACAGGG-3’ |
|  | Reverse | 5’-ACTTGTTGATGCCCATGCCT-3’ |
| *tlr4* | Forward | 5’-ACACATTGAACCAGGAGCTT-3’ |
|  | Reverse | 5’-TGTCCACGAAATGAAAGCCG-3’ |
| *tlr5* | Forward | 5’-GCAACAAACACCAGGACTCG-3’ |
|  | Reverse | 5’-CGCGCCCGTCTCTAATTGTA-3’ |
| *tlr7* | Forward | 5’-CGACGTTTCTCTGGCGAGTA-3’ |
|  | Reverse | 5’-AATTGCAGCGCAGGTCAATC-3’ |
| *tlr8a* | Forward | 5’-AAAGCTCAGCCTGGTGGAGAA-3’ |
|  | Reverse | 5’-AGGCTTCCTTTTTCTATGCGGT-3’ |
| *tlr8b* | Forward | 5’-AAGGCTACAACTGGGCTTGG-3’ |
|  | Reverse | 5’-GTCCAAGGTGACGGATGTGT-3’ |
| *tlr9* | Forward | 5’-ACTCCAGCTAATGGCAACCC-3’ |
|  | Reverse | 5’-CCCCGGTTCTCCAATCTCAC-3’ |
| *tab1* | Forward | 5’-CGAGGAGCAACAGCGATAGT-3’ |
|  | Reverse | 5’-ACTGGTGGTTTTGCACAGGA-3’ |
| *tbk1* | Forward | 5’-TGCGAGTGATTGGAGACGAC-3’ |
|  | Reverse | 5’-CTCCACAGATCCACCGTAGC-3’ |
| *trif* | Forward | 5’-CCAACCAAAGCATGACGACC-3’ |
|  | Reverse | 5’-AGGTAGCAGCGGAATAACGG-3’ |
| *tnf-α* | Forward | 5’-GGAGAGTTGCCTTTACCGCT-3’ |
|  | Reverse | 5’-CCTGGGTCTTATGGAGCGTG-3’ |
| *tnf-β* | Forward | 5’-GGTGTCGGGGGAGTTTATCA-3’ |
|  | Reverse | 5’-AAAAATGCAGCCACAACGCA-3’ |
| *nf-κB* | Forward | 5’-AGAGGACAATCTTCCGTCGC-3’ |
|  | Reverse | 5’-GGGTCTCTACGGGATAACGC-3’ |
| *bax* | Forward | 5’-TACTTTGCCTGTCGCCTTGT-3’ |
|  | Reverse | 5’-AGCGAGGAAAACTCCGACTG-3’ |
| *bcl2* | Forward | 5’-GCGGAGGGAACAACTCTGAA-3’ |
|  | Reverse | 5’-ATCCCGTAACACCCGGTAGA-3’ |
| *cox-2* | Forward | 5’-ATCATTCTTGGAGCGGTCTACT-3’ |
|  | Reverse | 5’-TACTCTGAGCGATGACATAGGC-3’ |
| *caspase-1* | Forward | 5’-TCAGCAAAGGAAATGGAT-3’ |
|  | Reverse | 5’-TTAGACGGCGGTAGACAT-3’ |
| *caspase-8* | Forward | 5’-CCAGGAACAAGGAGGCAGAC-3’ |
|  | Reverse | 5’-ATTGTGCCAGCCGAAGAGTT-3’ |
| *fadd* | Forward | 5’-GTGGTCTGGACGCGAAAGAG-3’ |
|  | Reverse | 5’-ACGGAGGGCCACTTTTTACC-3’ |
| *myd88* | Forward | 5’-ACGGCTAATCCCTGTCGTCT-3’ |
|  | Reverse | 5’-CAGATGGTCAGAAAGCGCAG-3’ |
| *il-4* | Forward | 5’-CTCGCATGCAGAATGACAGC-3’ |
|  | Reverse | 5’-TCGTTTTCCTTGATCGCCCA-3’ |
| *il-6* | Forward | 5’-GCAGTATGGGGGAACTATCCG-3’ |
|  | Reverse | 5’-TCCTGACCCCTTCAAATGCC-3’ |
| *il-8* | Forward | 5’-TCCTGGCATTTCTGACCATCAT-3’ |
|  | Reverse | 5’-ATGCGTCGGCTTTCTGTTTC-3’ |
| *il-10* | Forward | 5’-CGGGATATGGTGAAATGCAAGA-3’ |
|  | Reverse | 5’-AGAGCAAATCAAGCTCCCCC-3’ |
| *il-12* | Forward | 5’-CGACAATGAGGGCTCCTACC-3’ |
|  | Reverse | 5’-ATGTCTGTTCTCACCCCACG-3’ |
| *irf3* | Forward | 5’-CTACTGGGGTCTGTGCAAGC-3’ |
|  | Reverse | 5’-AAAATTGACCGGCCTACCAC-3’ |
| *irf5* | Forward | 5’-AACCTTCCAACTACCCAGCG-3’ |
|  | Reverse | 5’-TGAGCGACCATAGAAGCACC-3’ |
| *irf9* | Forward | 5’-GGCATCTGGAAGGATACGCT-3’ |
|  | Reverse | 5’-CAGGCCGAGATACTTCCCAC-3’ |
| *ripk1* | Forward | 5’-TGATGTACGAGAGCCACACG-3’ |
|  | Reverse | 5’-TACGAGCCACTTGCTTCCAG-3’ |
| *ripk3* | Forward | 5’-TTGTCCCGAGTGGCTGAAAA-3’ |
|  | Reverse | 5’-ACGGCTCCTTCCCAGTGATA-3’ |

**Table S2** Terms of GOids in GO enrichment analysis circle plot.

| **GOid** | **Term** |
| --- | --- |
| GO:0005622 | intracellular anatomical structure |
| GO:0002224 | toll-like receptor signaling pathway |
| GO:0043330 | response to exogenous dsRNA |
| GO:0015272 | ATP-activated inward rectifier potassium channel activity |
| GO:0060158 | phospholipase C-activating dopamine receptor signaling pathway |
| GO:0007286 | spermatid development |
| GO:0005164 | tumor necrosis factor receptor binding |
| GO:0009617 | response to bacterium |
| GO:0009615 | response to virus |
| GO:0005242 | inward rectifier potassium channel activity |
| GO:0010107 | potassium ion import across plasma membrane |
| GO:0000786 | nucleosome |
| GO:0042742 | defense response to bacterium |
| GO:0005125 | cytokine activity |
| GO:0006334 | nucleosome assembly |
| GO:0001889 | liver development |
| GO:0004867 | serine-type endopeptidase inhibitor activity |
| GO:0007267 | cell-cell signaling |
| GO:0005694 | chromosome |
| GO:0005615 | extracellular space |

**Table S3** Comparative table of the distribution of DEGs and all genes at GO Level2.

| **GO-classify1** | **GO-classify2** | **ALL** | **DEG** |
| --- | --- | --- | --- |
| biological process | biological adhesion | 2.470107 | 2.564103 |
|  | biological regulation | 36.36459 | 31.62393 |
|  | cell killing | 0.036711 | 0 |
|  | cellular component organization or biogenesis | 15.90099 | 6.837607 |
|  | cellular process | 58.84204 | 52.13675 |
|  | developmental process | 21.01951 | 12.82051 |
|  | establishment of localization | 12.38725 | 5.982906 |
|  | growth | 1.80407 | 2.564103 |
|  | immune system process | 4.982169 | 8.547009 |
|  | localization | 15.60206 | 7.692308 |
|  | locomotion | 3.796937 | 2.564103 |
|  | metabolic process | 39.50073 | 29.91453 |
|  | multi-organism process | 1.97189 | 6.837607 |
|  | multicellular organismal process | 22.15754 | 12.82051 |
|  | negative regulation of biological process | 6.702329 | 4.273504 |
|  | positive regulation of biological process | 7.058947 | 5.128205 |
|  | regulation of biological process | 33.75813 | 28.20513 |
|  | reproduction | 1.185232 | 2.564103 |
|  | reproductive process | 1.179987 | 2.564103 |
|  | response to stimulus | 25.25173 | 25.64103 |
|  | rhythmic process | 0.330396 | 0 |
|  | signaling | 19.15775 | 15.38462 |
|  | single-organism process | 0 | 0 |
| cellular component | cell | 54.16404 | 47.86325 |
|  | cell junction | 1.578561 | 0.854701 |
|  | cell part | 54.16404 | 47.86325 |
|  | extracellular matrix | 0.115377 | 0 |
|  | extracellular matrix part | 0.015733 | 0 |
|  | extracellular region | 5.244389 | 11.96581 |
|  | extracellular region part | 3.21481 | 7.692308 |
|  | macromolecular complex | 15.14055 | 10.25641 |
|  | membrane | 36.20726 | 35.04274 |
|  | membrane part | 32.89805 | 31.62393 |
|  | membrane-enclosed lumen | 4.452486 | 0.854701 |
|  | mitochondrion-associated adherens complex | 0 | 0 |
|  | nucleoid | 0.015733 | 0 |
|  | organelle | 33.88399 | 21.36752 |
|  | organelle part | 16.90266 | 9.401709 |
|  | symplast | 0 | 0 |
|  | synapse | 1.253409 | 0 |
|  | synapse part | 1.017411 | 0 |
|  | virion | 0.178309 | 0 |
|  | virion part | 0.178309 | 0 |
| molecular function | D-alanyl carrier activity | 0 | 0 |
|  | antioxidant activity | 0.267464 | 0 |
|  | binding | 51.15901 | 53.84615 |
|  | catalytic activity | 30.30732 | 26.49573 |
|  | channel regulator activity | 0.225509 | 0 |
|  | chemoattractant activity | 0 | 0 |
|  | chemorepellent activity | 0 | 0 |
|  | electron carrier activity | 0.152087 | 0 |
|  | enzyme regulator activity | 3.335431 | 3.418803 |
|  | metallochaperone activity | 0.026222 | 0 |
|  | molecular transducer activity | 6.628907 | 5.982906 |
|  | morphogen activity | 0 | 0 |
|  | nucleic acid binding transcription factor activity | 0 | 0 |
|  | nutrient reservoir activity | 0 | 0 |
|  | protein binding transcription factor activity | 0 | 0 |
|  | protein tag | 0.031466 | 0 |
|  | receptor activity | 0 | 0 |
|  | receptor regulator activity | 1.599538 | 5.128205 |
|  | structural molecule activity | 2.470107 | 0 |
|  | translation regulator activity | 0.062933 | 0 |
|  | transporter activity | 6.277533 | 3.418803 |

**Table S4** Distribution of up and down-regulated DEGs of GSTFTTTAER-vs-TNBS at KEGG Level2.

| **Classification-level2** | **Classification-level1** | **Type** | **Gene number** | **Percentage** |
| --- | --- | --- | --- | --- |
| Circulatory system | Organismal Systems | Up | 1 | 33.33 |
| Circulatory system | Organismal Systems | Down | 1 | 4.35 |
| Endocrine system | Organismal Systems | Down | 1 | 4.35 |
| Immune system | Organismal Systems | Up | 2 | 66.67 |
| Immune system | Organismal Systems | Down | 3 | 13.04 |
| Amino acid metabolism | Metabolism | Down | 1 | 4.35 |
| Carbohydrate metabolism | Metabolism | Down | 3 | 13.04 |
| Energy metabolism | Metabolism | Down | 1 | 4.35 |
| Lipid metabolism | Metabolism | Down | 3 | 13.04 |
| Infectious disease: bacterial | Human Diseases | Down | 1 | 4.35 |
| Infectious disease: viral | Human Diseases | Up | 1 | 33.33 |
| Infectious disease: viral | Human Diseases | Down | 3 | 13.04 |
| Folding, sorting and degradation | Genetic Information Processing | Down | 1 | 4.35 |
| Membrane transport | Environmental Information Processing | Down | 1 | 4.35 |
| Signal transduction | Environmental Information Processing | Down | 8 | 34.78 |
| Signaling molecules and interaction | Environmental Information Processing | Down | 7 | 30.43 |
| Cell growth and death | Cellular Processes | Up | 1 | 33.33 |
| Cell growth and death | Cellular Processes | Down | 2 | 8.7 |
| Cell motility | Cellular Processes | Down | 1 | 4.35 |


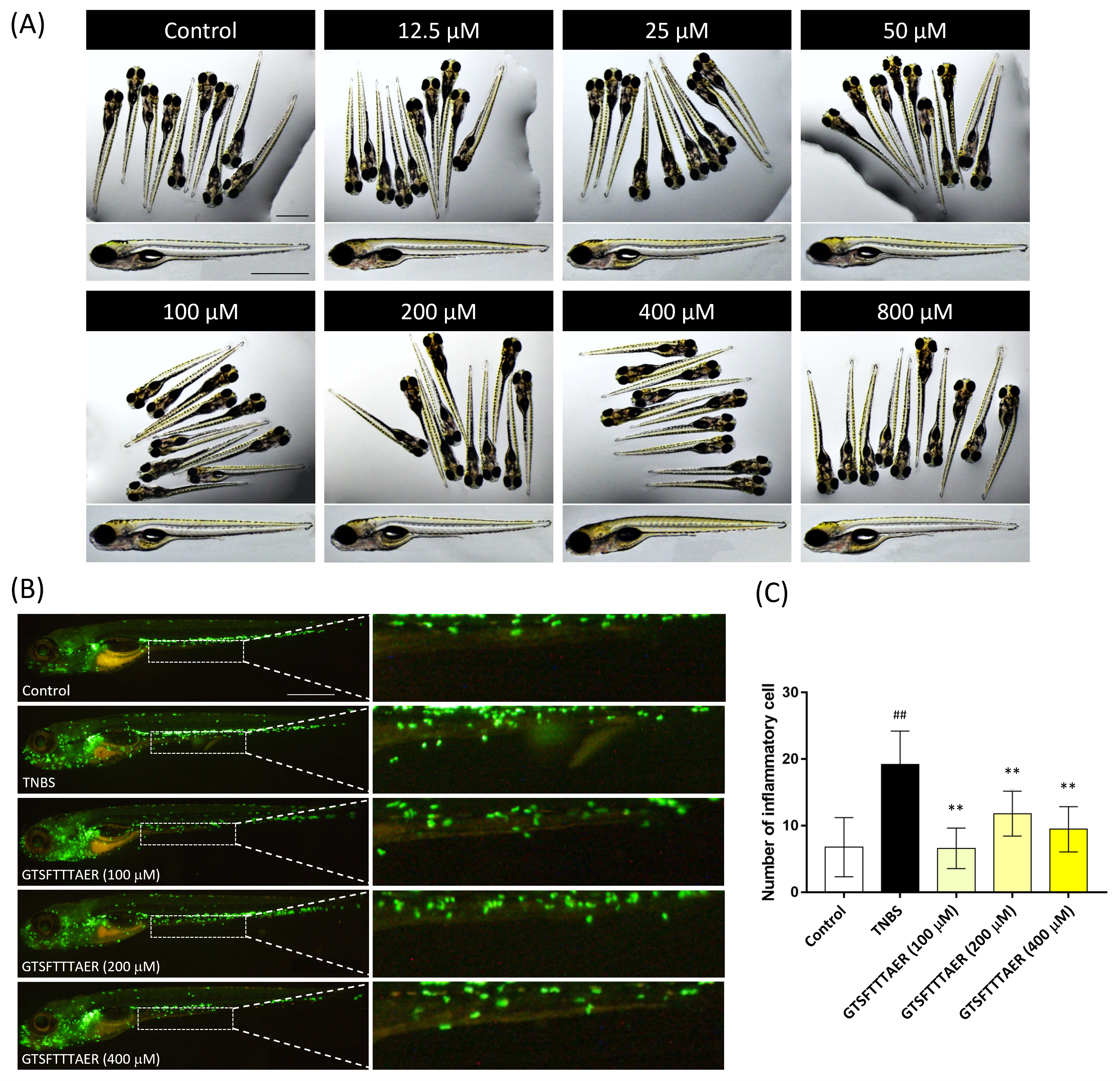


**Figure S1** Effects of different concentrations of peptide GTSFTTTAER in zebrafish. (A) Morphological diagram of the zebrafish. Scale bar is 1 mm. (B) Fluorescent immune cell images in Tg (*zlyz*: EGFP) zebrafish intestine. Scale bar is 500 µm. (C) Statistical analysis of the number of immune cells in zebrafish intestine. ^##^p < 0.01 versus the control group; ^**^p < 0.01 versus the TNBS group.


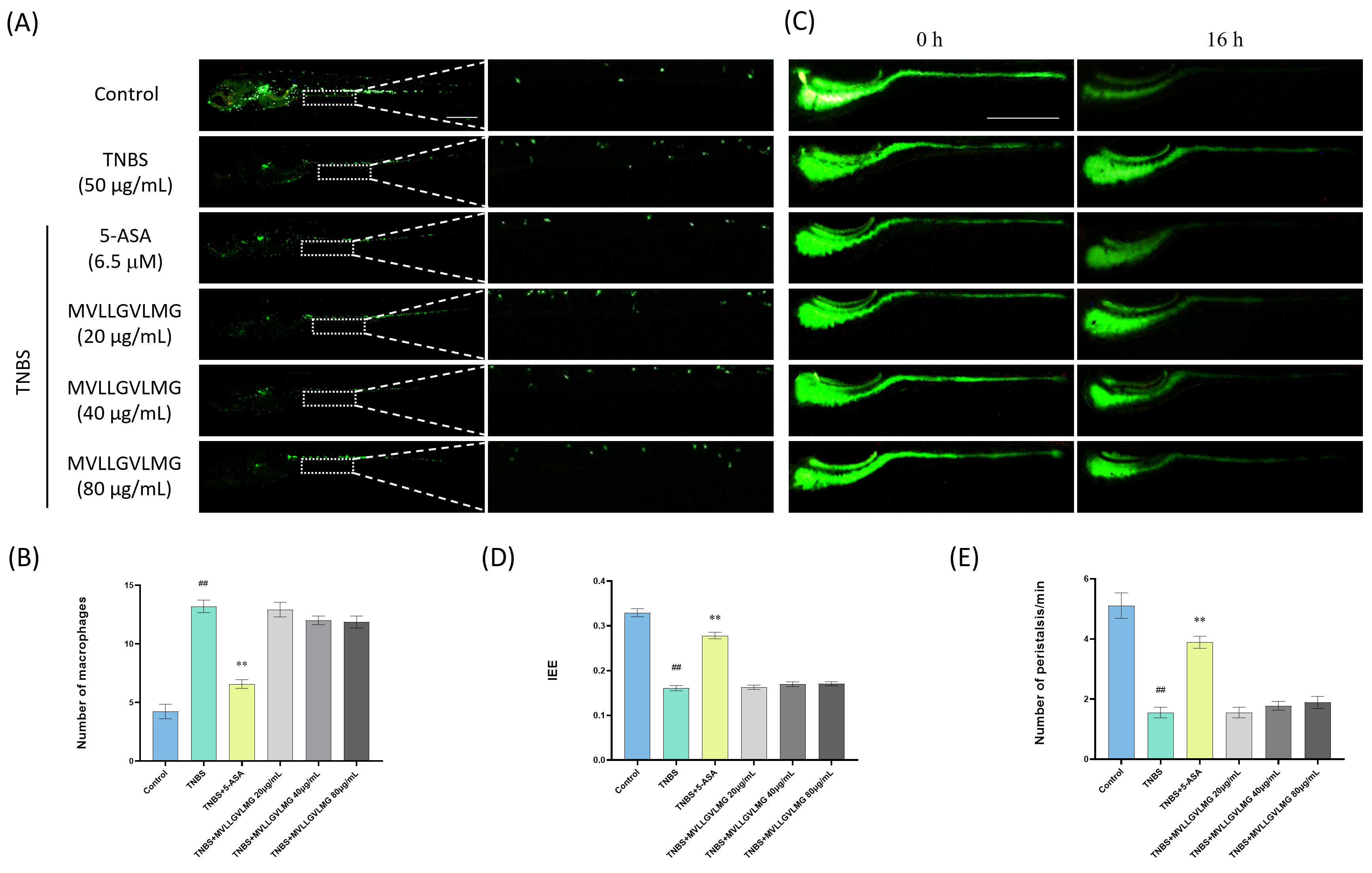


**Figure S2** Effects of the peptide MVLLGVLMG on TNBS-induced zebrafish larvae. (A) Fluorescent immune cell images in Tg (*zlyz*: EGFP) zebrafish intestine. Scale bar is 500 µm. (B) Statistical analysis of the number of immune cells in zebrafish intestine. (C) Representative fluorescence images of wild-type zebrafish intestine. Scale bar is 500 µm. (D-E) Statistical analysis of the intestinal efflux efficiency and the frequency of intestinal peristalsis. ^##^p < 0.01 versus the control group; ^**^p < 0.01 versus the TNBS group.
